# Global efficiency of the motor network is decreased in Parkinson's disease in comparison with essential tremor and healthy controls

**DOI:** 10.1002/brb3.2178

**Published:** 2021-07-24

**Authors:** Natalia Pelizari Novaes, Joana Bisol Balardin, Fabiana Campos Hirata, Luciano Melo, Edson Amaro, Egberto Reis Barbosa, João Ricardo Sato, Ellison Fernando Cardoso

**Affiliations:** ^1^ Neurology Universidade de São Paulo São Paulo Brazil; ^2^ Hospital Israelita Albert Einstein São Paulo Brazil; ^3^ Radiology Universidade de São Paulo São Paulo Brazil; ^4^ Universidade Federal do ABC Santo André Brazil; ^5^ Hôpital du Valais Sion Switzerland

**Keywords:** functional connectivity, functional magnetic resonance imaging, graph theory, movement disorders, tremor physiology

## Abstract

**Background:**

Graph theory (GT) is a mathematical field that analyses complex networks that can be applied to neuroimaging to quantify brain's functional systems in Parkinson's disease (PD) and essential tremor (ET).

**Objectives:**

To evaluate the functional connectivity (FC) measured by the global efficiency (GE) of the motor network in PD and compare it to ET and healthy controls (HC), and correlate it to clinical parameters.

**Methods:**

103 subjects (54PD, 18ET, 31HC) were submitted to structural and functional MRI. A network was designed with regions of interest (ROIs) involved in motor function, and GT was applied to determine its GE. Clinical parameters were analyzed as covariates to estimate the impact of disease severity and medication on GE.

**Results:**

GE of the motor circuit was reduced in PD in comparison with HC (*p* .042). Areas that most contributed to it were left supplementary motor area (SMA) and bilateral postcentral gyrus. Tremor scores correlated positively with GE of the motor network in PD subgroups. For ET, there was an increase in the connectivity of the anterior cerebellar network to the other ROIs of the motor circuit in comparison with PD.

**Conclusions:**

FC measured by the GE of the motor network is diminished in PD in comparison with HC, especially due to decreased connectivity of left SMA and bilateral postcentral gyrus. This finding supports the theory that there is a global impairment of the motor network in PD, and it does not affect just the basal ganglia, but also areas associated with movement modulation. The ET group presented an increased connectivity of the anterior cerebellar network to the other ROIs of the motor circuit when compared to PD, which reinforces what it is known about its role in this pathology.

## Graphical abstract

This study is a graph theory evaluation of the functional connectivity measured by the global efficiency of the motor network in Parkinson's disease (PD) in comparison with essential tremor (ET) and healthy controls (HC). We have found that global efficiency is decreased in PD when compared to ET and HC. Areas that contributed to it were the left SMA and bilateral postcentral gyrus.

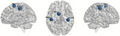

## 1 INTRODUCTION

Parkinson's disease (PD) and essential tremor (ET) are the most prevalent movement disorders in adults and the most frequent causes of tremor. Although many studies have contemplated PD and ET pathophysiology, the mechanisms that lead to neuronal degeneration and loss of functionality are not completely understood. Furthermost, they involve not only isolated regions, but also the circuits required for functional integrative processes, specially the cerebello‐thalamo‐cortical circuit and the basal ganglia.

Neuroimaging techniques have been a growing field of study concerning neurodegenerative diseases, not only focusing on diagnosis but also in the attempt to elucidate neuropathology and its mechanisms. Several fMRI studies have already been performed in order to investigate the motor network in PD and ET, mostly focusing on pairwise comparisons such as seed‐to‐voxel and voxel‐to‐voxel connectivity. Graph theory (GT) is the mathematical field that analyses complex networks and has been applied to neuroimaging data to quantify brain's functional systems (Bullmore & Sporns, 2009). One of the measures of GT is global efficiency (GE), which is a frequently used metric to study integration within brain networks. GE is mathematically expressed as the inverse of the shortest path length, and it reflects effective information transfer within a network. Only a small number of studies regarded GE in PD and ET, with conflicting results. While most studies in PD showed decreased GE of the motor network (Abbasi et al., 2018; Cai et al., 2019; Suo et al., 2017; Vriend et al., 2018), some found no abnormalities (Hou et al., 2018) or even an increased GE (Fang et al., 2017; Guan et al., 2019; Nigro et al., 2019).

Concerning PD, there is a hypothesis that the dopamine deficit in the striatum‐thalamus‐cortical system is somehow compensated by hyperactivity of the cerebellum‐thalamus‐cortical circuit and that would lead to tremor. Functional Magnetic Resonance Imaging (fMRI) studies correlated tremor with activity in putamen, pallidum, cerebellum, ventrointermediate nucleus of the thalamus (VIM), motor and premotor cortices (Helmich et al., 2011). The cerebellum‐thalamo‐cortical circuit seems to be responsible for tremor amplitude, while basal ganglia such as subthalamic nucleus (STN) and internal globus pallidus (GPi) would be responsible for tremor initiation, since they oscillate at the same tremor frequency. The decrease in dopaminergic projections would increase GPi activity, which would result in hyperpolarization of neurons that receive projections from GPi in the thalamus (Albin et al., 1989). This increase of inhibition from GPi over thalamic nucleus would, by its turn, raise the chance of generating activity in rhythmic neuronal groups in the thalamus (Caligiore et al., 2016). This is known as the “dimmer‐switch” hypothesis, in which the basal ganglia would generate the trigger for tremor and the cerebellum‐thalamus‐cortical circuit would modulate its amplitude (Helmich et al., 2013). This theory was later revisited by Helmich (2018). In the thalamus, tremor‐related activity was localized in the Ventrolateral nucleus in its posterior part (VLp), which receives afferents from the cerebellum. In the cerebellum, tremor was related to lobules V and VI, which is the sensorimotor portion. In the cerebral cortex, tremor was associated with activity in primary motor cortex and premotor cortex, and also in the somatosensory cortex, with an important role in tremor amplitude. Basal ganglia did not relate to tremor amplitude, but to onset of tremor episodes.

Previous fMRI studies have shown that motor network is affected in ET, with diminished functional connectivity between cerebellum and cortex that correlates with symptoms intensity, and also an increased connectivity between right cerebellar lobules I‐IV and left thalamus (Buijink et al., 2015). These findings suggest that cerebello‐dentato‐thalamic activity and cerebello‐cortical connectivity are disrupted in ET. There is large evidence of cerebellar dysfunction in ET: cerebellar stroke can ameliorate ipsilateral tremor (Dupuis et al., 2010); spectroscopy studies showed reduction in N‐acetylaspartate in cerebellum of ET patients (Pagan et al., 2003); voxel‐based morphometry showed cerebellar atrophy (Bagepally et al., 2012); anatomopathological studies showed a loss of Punkinje cells, dendritic edema and an increase in their axonal ramifications (Lin et al., 2014). In line with that, one open‐label study performed repetitive TMS of the cerebellum and showed that not only the clinical tremor scores improved but also the functional connectivity of the cerebello‐thalamo‐cortical network was reestablished (Popa et al., 2013). Moreover, it has been long known that ventral intermediate (VIM) DBS partially restores the cerebello‐thalamo‐cortical pathway and reduces tremor (Molnar et al., 2004). A recent systematic review of cerebellar neuromodulation through different techniques in movement disorders suggests that cerebellar modulation improved tremor in ET (França et al., 2018).

According to what is known about PD pathophysiology, we hypothesized that there would be a decreased global efficiency of the motor network in PD patients when compared to controls and to ET patients. This decrease would be related to clinical parameters such as tremor scores. We also hypothesized that ET patients would show different connectivity patterns from controls, specially concerning the cerebellum, possibly with increased connectivity to some areas in the motor network. Since our study has a large number of participants, great quality equipment, short repetition time (TR), and used very strict preprocessing parameters and conservative statistical analysis, it is hoped to contribute to clarify some of these controversies.

## 2 METHODS

### 2.1 Patient selection

Patients treated at the Movement Disorders Clinic of *Hospital das Clínicas da Universidade de São Paulo* and *Associação Brasil Parkinson* were invited to participate in this study, after its approval by the local ethics committee (Online registration n° 8197, CAPPesq n° 0700/11). All individuals signed the Term of Consent. Inclusion criteria for PD patients were as follows: to have idiopathic PD, according to the UK Brain Bank Criteria (Hughes et al., 1992), and Hoehn & Yahr I or II (Hoehn & Yahr, 1967); for ET patients, the diagnostic criteria established by the Movement Disorders Society (Deuschl et al., 1998), absence of parkinsonism and exclusion of medication‐induced tremor. For healthy controls, criteria included absence of neurological diseases, and age pairing with the patients' group. Tables 1 and 2 summarize the clinical and epidemiological data of patients' groups and controls. Exclusion criteria were impossibility of performing an MRI, a severe movement disorder that would impair imaging analysis, and intracranial structural abnormalities.

**TABLE 1:** Clinical and epidemiological data of all subjects

Groups	DP	ET	HC
Number of subjects	54	18	31
Age	64.1	68.7	63.3
Gender (%M)	76	33.3	24.1

**TABLE 2:** Clinical and demographical characteristics of Parkinson's subgroups

Groups	DP	DPT	DPAR
Number of subjects	54	19	24
Age Mean (*SD*)	64.1 (11.5)	67.3 (9.9)	64 (14.1)
Gender (%M)	76		
UPDRS Mean (*SD*)	50.6 (16.5)	49.5 (18.9)	51.4 (14.6)
Disease duration Mean (*SD*)	5.8 (5.5)	5.8 (5.1)	6 (5.6)
LED Mean (*SD*)	574.0 (401.6)	450.4 (241.8)	671.8 (475.5)

PD patients were classified by the UPDRS (Unified Parkinson's Disease Rating Scale) (Goetz et al., 2008) and were submitted to MRI scans while under effect of Levodopa (ON state). This decision could pose questions whether the results of the fMRI would be due to the physiopathological changes in PD brain or due to medication effects (Tahmasian et al., 2015). However, many patients do not tolerate the MRI scan in OFF state (without effect of medication), due to tremor, axial rigidity, pain, and discomfort. Moreover, analysis of fMRI during the ON state would be closer to patient daily activities scenario. The Levodopa Equivalent Dose (LED) was calculated for each subject using the Birmingham University conversion formula, and it was used as a covariate in statistical analysis. Patients were subdivided into two subgroups—tremulant‐predominant group (PDT) and akinetic‐rigid group (PDAR), according to criteria proposed by Jankovic (Jankovic et al., 1990). We calculated the average between the sum of the UPDRS items associated with tremor ( (16, 20 and 21) and the sum of items associated to rigidity, gait and postural instability (13, 14, 15, 29 and 30). Patients with average equal or below 1 were classified as PDAR and patients with average above or equal to 1.5 were classified as PDT.

### 2.2 Image acquisition

fMRI scans were performed at Siemens Trio Scanner 3.0T MR, with 32 channels head coil and gradient of 45 mT/m. Whole brain volumes were acquired by the echo‐planar imaging (EPI) multi‐band accelerated sequence with repetition time (TR) of 600 ms (Moeller et al., 2010). BOLD‐sensitive images were acquired on T2*‐weighted sequences. Acquisition parameters were as follows: 40 axial cuts, slice thickness = 2.5 mm, TE = 31 ms, NEX = 1, Flip angle = 90°, Bandwidth = 2290Hz/px, FOV = 210 mm, matrix size = 84 × 84, voxel dimension = 2.5 mm × 2.5 mm × 2.5 mm. All images were acquired while patients were lying still with eyes closed (resting state). For coregistration and normalization, structural images with high resolution were also acquired (MP2RAGE).

### 2.3 Image preprocessing

Data were preprocessed and analyzed using the CONN toolbox version 17.b (Whitfield‐Gabrieli & Nieto‐Castanon, 2012), with a standard MNI152 pipeline and parameters. Preprocessing steps included realignment and unwarping, slice‐timing correction, segmentation, normalization, outlier detection, and smoothing. Nuisance variables were based on scan motion censuring (discarding volumes with displacement >2mm and global‐signal *z*‐value >9; no subjects were excluded), 12 realignment parameters, white matter, and cerebrospinal fluid signals. Band‐pass filtering (0.008–0.09 Hz) and nuisance variables were regressed out using a simultaneous band‐pass approach (Hallquist et al., 2013).

### 2.4 ROI selection

**FIGURE 1:**
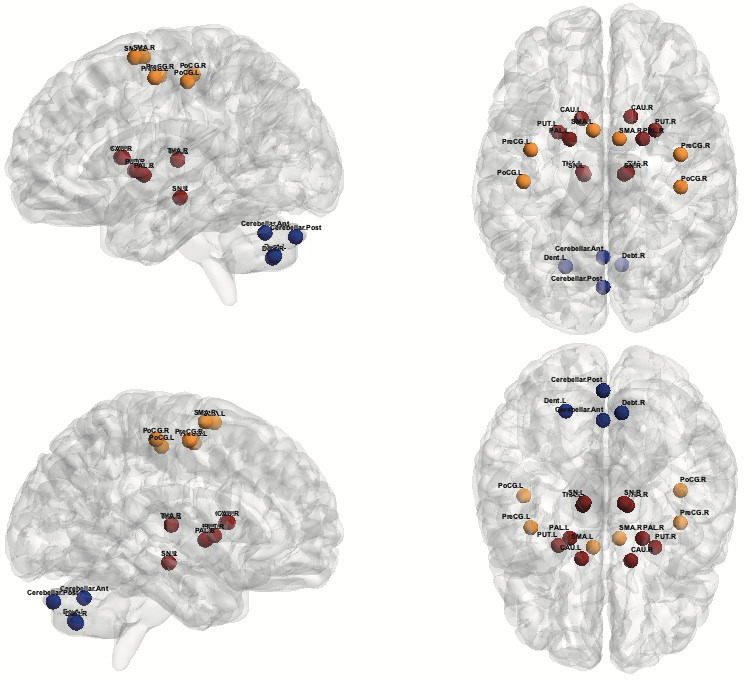
Regions of interest (ROIs): In orange, cortical ROIs, bilaterally (Precentral gyrus, Postcentral gyrus, Supplementary Motor Area); in red, substantia nigra and subcortical ROIs (caudate, putamen, pallidum, thalamus); in blue, cerebellar ROIs (cerebellar anterior network, cerebellar posterior network, and dentate nucleus right and left). Note: Colors were used just for didactic purposes; they do not infer degree of connectivity

Regions of interest (ROIs) were determined based on the Automated Anatomical Labeling Atlas (AAL) (Tzourio‐Mazoyer et al., 2002) and Harvard‐Oxford Atlas (Frazier et al., 2005). We manually created ROIs for the dentate nucleus and substantia nigra (Dimitrova et al., 2006; Menke et al., 2010) using MatlabR2018b. They were chosen due to their relevance to the motor network and that would be somehow implicated in the tremor physiopathology: dentate nucleus, cerebellar networks anterior and posterior, substantia nigra, thalamus, caudate, putamen, pallidum, precentral gyrus, postcentral gyrus, and supplementary motor area. ROIs are displayed in Figure 1.

### 2.5 Connectivity estimation and analysis

Graph theory analyses were also computed using the CONN toolbox. We focused on the global efficiency metric since it has been shown to be one of the most robust measures to assess integration properties of brain networks (Duda et al., 2014). It reflects effective information transfer within a network of nodes (i.e., ROIs) and edges (i.e., correlations or “paths” between nodes) (Watts & Strogatz, 1998). The unweighted ROI‐to‐ROI correlation matrices of the motor network (one for each participant) were first submitted to a threshold at a cost value of *k* = 0.15. Global efficiency indices were thresholded at p‐FDR < 0.05 in a two‐sided analysis based on correlation scores. Independent sample *t* tests were performed to examine differences between groups on global efficiency scores (PD, ET, and HC), two at a time. Statistical analyses were conducted by using JASP with *p* <.05, one‐tailed.

The relation between global efficiency and clinical parameters (UPDRS, tremor scores alone from UPDRS and levodopa equivalent dose) in the PD group was tested using Spearman correlation. Differences in these associations between patients' subgroups were further tested using separate ANCOVAs with group as the fixed factor and global efficiency as the dependent variable.

## 3 RESULTS

### 3.1 Differences between clinical diagnosis on the global efficiency of the motor network

**FIGURE 2:**
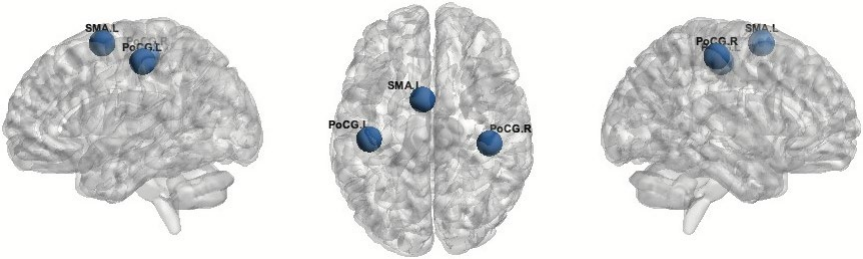
Results of global efficiency of the motor network in the PD group versus HC group. Blue areas are left SMA, Postcentral gyrus right and left

**FIGURE 3:**
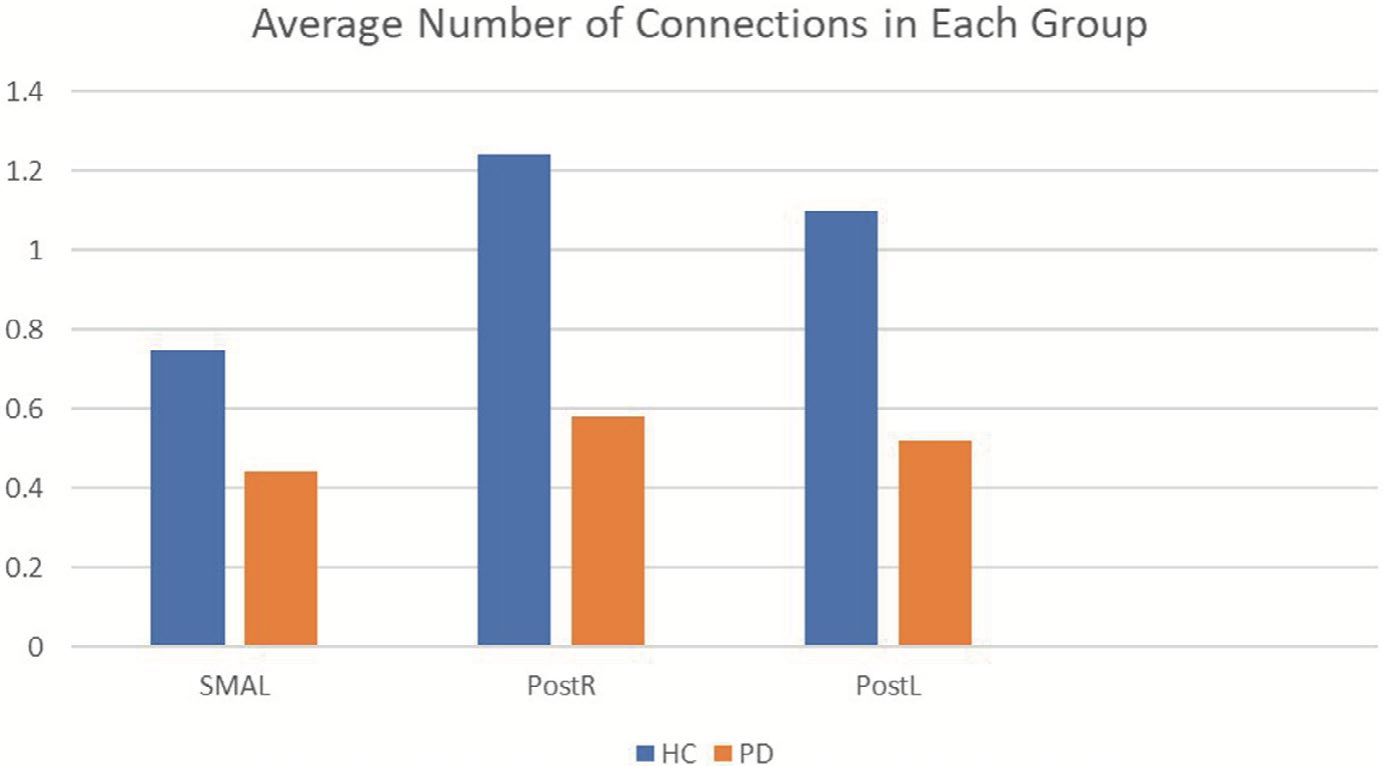
Graphic representation of the average number of connections in each group. On the *x*‐axis, the ROIs that were responsible for the difference in GE between the two groups. On the y‐axis, the average number of connections in each group

Global efficiency of the motor network was reduced in PD Group when compared to HC (*p* = .042). The average GE of the network in PD patients was 0.0231, versus 0.0297 for controls. Left SMA had the lowest GE (0.022 FDR‐corrected) in the PD group, followed by Postcentral gyrus left (0.0496 FDR‐corrected) and Postcentral gyrus right (0.0496 FDR‐corrected) (Figure 2). In order to better interpret, these results of GE—particularly what is the meaning of the ROIs displayed on the CONN output results—we calculated for each ROI of the network the average number of connections obtained by the adjacency matrix, as shown in Figure 3. These values confirm that the average number of connections is larger in the HC group. Therefore, a reduced global efficiency in PD is possibly driven by a lower number of connections of these three nodes (left SMA, Left and Right Postcentral gyrus) within the motor network in this group. The difference of global efficiency of the motor network was also found when comparing the PDAR subgroup to HC (*p* .035), but not when comparing the PDT group to HC (*p* .092). No difference was found between the two subgroups of PD in our study (*p* .753).

For essential tremor, there was no difference in GE of the motor network when compared to HC (*p* .089) nor to PD (*p* .976), and neither to PD subgroups (PT × ET *p* .914, PDAR × ET *p* .838). However, there was an increase in the GE of the anterior cerebellar network to the other ROIs of the motor network in the ET group, when compared to the PD group (*p* .001).

### 3.2 Relation to clinical characteristics

Relations to clinical characteristics were examined for PD group and both subgroups, separately. UPDRS Scores did not correlate to GE in PD group or subgroups. Tremor scores alone correlated positively with GE in both PD subgroups, but were not statistically different between them (*p* .97). Tremor scores correlated positively to the GE of the left substantia nigra in both subgroups and were statistically different between them (*p* = .001), being greater in the PDAR group.

LED was also used as a covariate to study the possible influence of the medication on GE, but no statistically significant result was found in PD group and subgroups. In order to exclude motor artifacts as possible confounders for connectivity analysis, we evaluated the correlations of mean motion and GE and found no relation between them. (More information on covariates analysis can be found on Appendix S1).

## 4 DISCUSSION

According to our previous hypothesis, motor network connectivity measured by the GE is decreased in Parkinson's disease in comparison with healthy controls. Areas that most contributed to this diminished connectivity were left SMA and bilateral postcentral gyrus. Tremor scores correlated positively to the GE of the network in PD and influenced the connectivity of the left substantia nigra differently in both groups, especially in the PDAR group. Contrary to our expectations, the global efficiency of the motor network was not different between PD subgroups, possibly because our sample of patients was rather homogeneous, or perhaps because the sample of subgroups was not as numerous.

Other studies have also evaluated the GE of the motor network, with conflicting outcomes. In accordance with our results, one study analyzed longitudinally 16 PD patients and showed decreased connectivity between the SMA, pre‐ and postcentral gyri when compared to controls (Tuovinen et al., 2018). In addition, another group with a larger number of participants demonstrated that GE is decreased in PD patients when compared to HC, and such decrease correlates with cerebrospinal fluid biomarkers (CSF), such as alfa‐synuclein, Aβ_42_, and total TAU. The data suggest that functional imaging and measurement of CSF biomarkers together can bring a better understanding of PD pathogenesis (Abbasi et al., 2018). However, Hou et al. (2018) and Berman et al. (2016) found no global level abnormalities, while Guan et al. (2019) have found increased GE, which correlated positively to iron accumulation in the inferior SN.

We know SMA plays a direct role in movement control thru direct projections to the spinal cord, and also throughout interactions with other structures to help control postural stability, bimanual coordination, sequences of movements, and initiation of internally generated movement. Previous studies have reported SMA connectivity disruption in PD, with controversial results. A few studies showed enhanced connectivity of the SMA to the putamen and amygdala (Yu et al., 2013) and increased pre‐SMA activation in early PD patients while performing self‐initiated movements (Eckert et al., 2006). Another study also using graph theory to measure network interactions showed decreased connectivity in SMA, left dorsolateral prefrontal cortex, and left putamen (Wu et al., 2009). The latter study compared PD ON and OFF medication, and this decreased connectivity of SMA persisted on both states. Other methods such as PET and SPECT have also shown decreased SMA activation in comparison with controls during internally triggered movements (Jahanshahi et al., 1995), and increased activation in SMA in PD patients under DBS (Ceballos‐Baumann et al., 1999) or apomorphine (Jenkins et al., 1992). Therefore, SMA may play an important role in PD physiopathology and should be considered as a target in neuromodulation studies.

The postcentral gyrus is the primary sensorial area, but we wondered if it could also be involved in tremor modulation because of its peripheral proprioceptive information for the movement feedback loops. Another question is if its strong connections with the thalamus would play a role in tremor control. One previous research has shown diminished activation in somatosensory cortex in PD after tactile stimulation (Nelson et al., 2018). A systematic review and meta‐analysis englobed thirty studies, with a total of 854 PD patients, and emphasized the role of postcentral gyrus as a critical region in PD. They have found an increased functional connectivity in postcentral gyrus in PD compared to healthy controls (Ji et al., 2018).

The fact that SN connectivity was influenced differently by tremor scores in both subgroups of PD could raise the question to whether this area would be implicated in the differences of clinical manifestation between them. Furthermore, contrary to our expectations, global efficiency was positively correlated to tremor scores. One study found no relation of GE and motor severity (Fang et al., 2017), and another research using apomorphine described a reduction of tremor mirrored by an increase in overall connectivity strength, but they did not use the same network measures as we did (Nigro et al., 2019). Therefore, the biological interpretation of these values is not straightforward.

Regarding ET, the fact that the connectivity of the anterior cerebellar network was increased to the other regions of the motor network when compared to PD reinforces its role in the pathogenesis of tremor is this disease. The cerebellum integrates multimodal sensorimotor inputs from the cortex, vestibular nuclei, and spinal cord and modulates the information thru outputs to the cerebral cortex, across the cerebello‐thalamo‐cortical tract, so that movement is harmonic and smooth. (Maas et al., 2020) One fMRI study using graph theory analysis found disruption in the efficiency of the overall brain functional network in ET, involving multiple areas of the brain. In the global level, ET patients exhibited lower small‐worldness values than HC, and at the regional level, showed higher values of GE in several cortical and cerebellar areas (Benito‐León et al., 2019).

A limitation of our study, as already mentioned and justified, is that PD patients were scanned under the effect of Levodopa to minimize motion and discomfort during scanning. There have been previous reports in the literature suggesting that levodopa could be a confounding factor for connectivity analyses (Tahmasian et al., 2015), with a tendency to “normalize” network measures such as local efficiency (Berman et al., 2016). A network study comparing patients in ON and OFF states showed that levodopa normalized degree centrality abnormalities specially in occipital regions and postcentral gyrus (Zhong et al., 2019). However, we used LED as a covariate to study its effect on the GE of the network and found no correlation. Another limitation was the small sample of ET patients, possibly due to the fact that patients were recruited at a tertiary movement disorder center, which tends to refer nondisabling cases to secondary services.

We believe that one of the strengths of our study is that we used very strict preprocessing parameters in order to control for motion and artifacts. Moreover, our statistical analysis was very conservative, according to current tendencies of the international neuroimaging community. Another strong point, besides strict preprocessing and statistics, was our large sample, high‐quality equipment, and very short TR (0.6 s).

Therefore, our current work demonstrates decreased connectivity of the motor system in PD in comparison with controls, giving a relevant contribution to a growing body of studies on the network disruption in Parkinson's disease and essential tremor, and hopefully might contribute to the development of new strategies of intervention such as DBS or TMS to better treat PD and ET.

## CONFLICT OF INTEREST

Authors report no conflicts of interest.

### PEER REVIEW

The peer review history for this article is available at https://publons.com/publon/10.1002/brb3.2178.

## Supporting information

Appendix S1

## Data Availability

The data that support the findings of this study are available upon request.
