# Supplementary material for: Global efficiency of the motor network is decreased in Parkinson's disease in comparison with essential tremor and healthy controls
Source: Brain Behav. 2021 Jul 24;11(8):e02178. doi: 10.1002/brb3.2178 (PMC8413813; doi:10.1002/brb3.2178)

Supplement 1

Covariates Analysis

The graphics below show the correlations amongst Global Efficiency and Image Acquisition Parameters (mean motion) and Clinical Characteristics such as UPDRS, tremor scores and Levodopa equivalent dose (Spearman correlation), for the PD group.


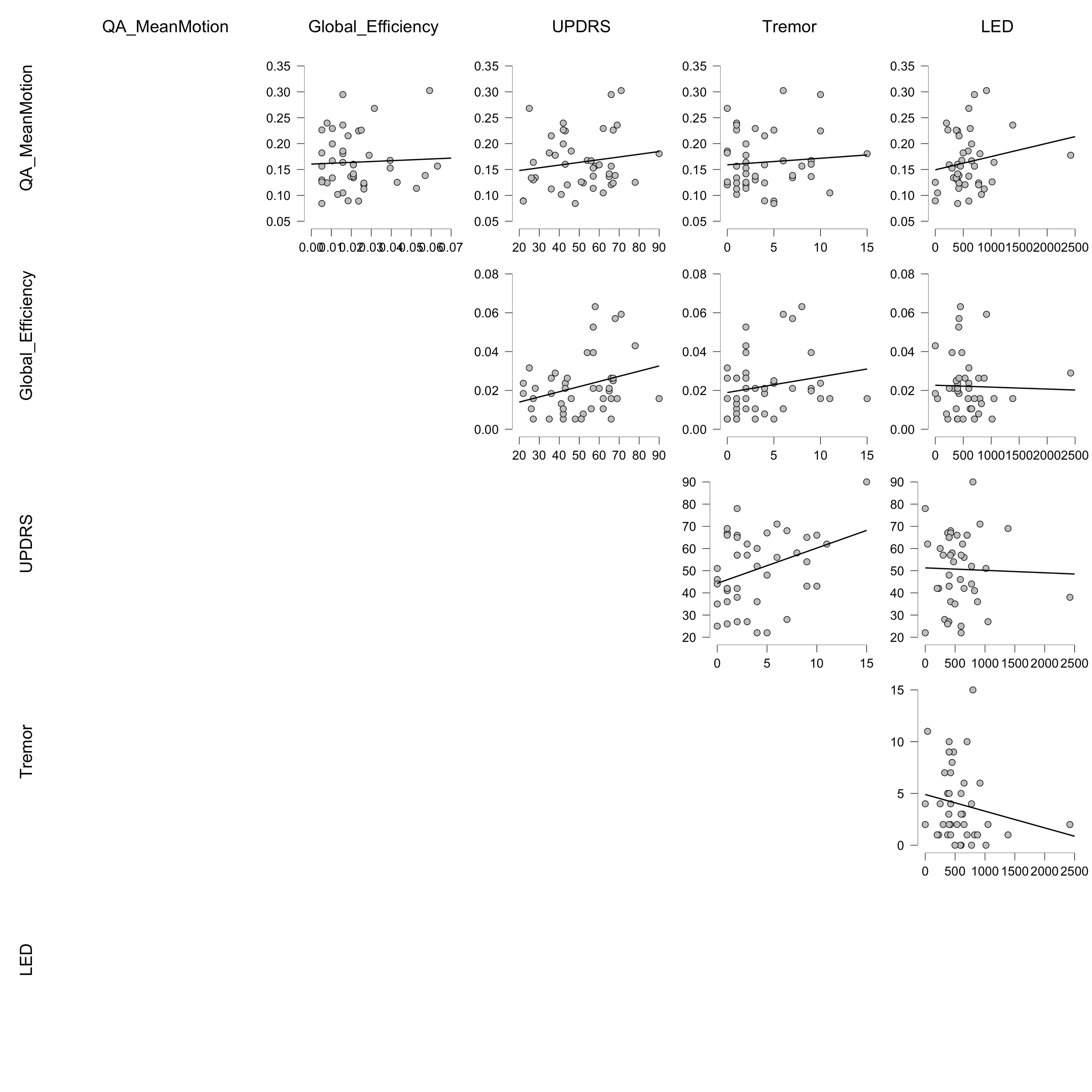


The graphics below represent the correlation of the tremor scores (x axis) with the global efficiency (y axis) on the two subgroups of PD. First graphic represents the PDAR subgroup, and the second represents PDT group

The table below shows that the association of tremor scores and global efficiency is not different in the PD subgroups (p 0.097).

| **ANCOVA – Global Efficiency** | | | | | | | | | | | |
| --- | --- | --- | --- | --- | --- | --- | --- | --- | --- | --- | --- |
| **Cases** | | **Sum of Squares** | | **df** | | **Mean Square** | | **F** | | **p** | |
| Subgroup |  | 3.934e -5 |  | 1 |  | 3.934e -5 |  | 0.185 |  | 0.669 |  |
| Tremor |  | 0.001 |  | 1 |  | 0.001 |  | 5.314 |  | 0.027 |  |
| Subgroup ✻ Tremor |  | 6.140e -4 |  | 1 |  | 6.140e -4 |  | 2.888 |  | 0.097 |  |
| Residual |  | 0.008 |  | 39 |  | 2.126e -4 |  |  |  |  |  |
|  | | | | | | | | | | | |

The graphics below represent the correlation of the global efficiency of the left substantia nigra (y axis) and tremor scores (x axis) on the two subgroups of PD. The first graphic represents the PDAR group, and the second represents the PDT group (p 0.001).

| **ANCOVA - GE_Subst Nigra L** | | | | | | | | | | | |
| --- | --- | --- | --- | --- | --- | --- | --- | --- | --- | --- | --- |
| **Cases** | | **Sum of Squares** | | **df** | | **Mean Square** | | **F** | | **p** | |
| Subgroup |  | 0.003 |  | 1 |  | 0.003 |  | 0.219 |  | 0.642 |  |
| Tremor |  | 0.250 |  | 1 |  | 0.250 |  | 19.243 |  | < .001 |  |
| Subgroup ✻ Tremor |  | 0.155 |  | 1 |  | 0.155 |  | 11.903 |  | 0.001 |  |
| Residual |  | 0.506 |  | 39 |  | 0.013 |  |  |  |  |  |
|  | | | | | | | | | | | |

The table above shows that effect of tremor interferes differently in the connectivity of the left substantia nigra between the two groups, being more relevant in the PDAR group. However, if we exclude the subject who showed an abnormally high GE in the PDAR group, this difference would disappear, as shown in table below.

| **ANCOVA - GE_Subst Nigra L** | | | | | | | | | | | |
| --- | --- | --- | --- | --- | --- | --- | --- | --- | --- | --- | --- |
| **Cases** | | **Sum of Squares** | | **df** | | **Mean Square** | | **F** | | **p** | |
| Subgroup |  | 0.001 |  | 1 |  | 0.001 |  | 0.109 |  | 0.743 |  |
| Tremor |  | 0.026 |  | 1 |  | 0.026 |  | 2.659 |  | 0.111 |  |
| Subgroup ✻ Tremor |  | 0.006 |  | 1 |  | 0.006 |  | 0.627 |  | 0.433 |  |
| Residual |  | 0.374 |  | 38 |  | 0.010 |  |  |  |  |  |
|  | | | | | | | | | | | |
|  | | | | | | | | | | | |

The following plots correspond to the PDT group covariates correlation analyses.

## Correlation

| **Spearman's Correlations** | | | | | | | | | | | | | | | | | |  |
| --- | --- | --- | --- | --- | --- | --- | --- | --- | --- | --- | --- | --- | --- | --- | --- | --- | --- | --- |
| **Variable** |  | | **Global_Efficiency** | | **UPDRS** | | | **Tremor** | | | **LED** | | | **QA_MeanMotion** | | | |  |
| Global_Efficiency |  | Spearman's rho |  | — | |  |  | |  |  | |  |  | |  |  |  | |
|  |  | p-value |  | — | |  |  | |  |  | |  |  | |  |  |  | |
| UPDRS |  | Spearman's rho |  | 0.150 | |  | — | |  |  | |  |  | |  |  |  | |
|  |  | p-value |  | 0.540 | |  | — | |  |  | |  |  | |  |  |  | |
| Tremor |  | Spearman's rho |  | 0.174 | |  | 0.575 | |  | — | |  |  | |  |  |  | |
|  |  | p-value |  | 0.475 | |  | 0.010 | |  | — | |  |  | |  |  |  | |
| LED |  | Spearman's rho |  | -0.057 | |  | 0.136 | |  | 0.156 | |  | — | |  |  |  | |
|  |  | p-value |  | 0.816 | |  | 0.579 | |  | 0.523 | |  | — | |  |  |  | |
| MeanMotion |  | Spearman's rho |  | 0.168 | |  | 0.409 | |  | 0.275 | |  | 0.421 | |  | — |  | |
|  |  | p-value |  | 0.492 | |  | 0.082 | |  | 0.254 | |  | 0.072 | |  | — |  | |
|  | | | | | | | | | | | | | | | | | |  |

### Correlation plot


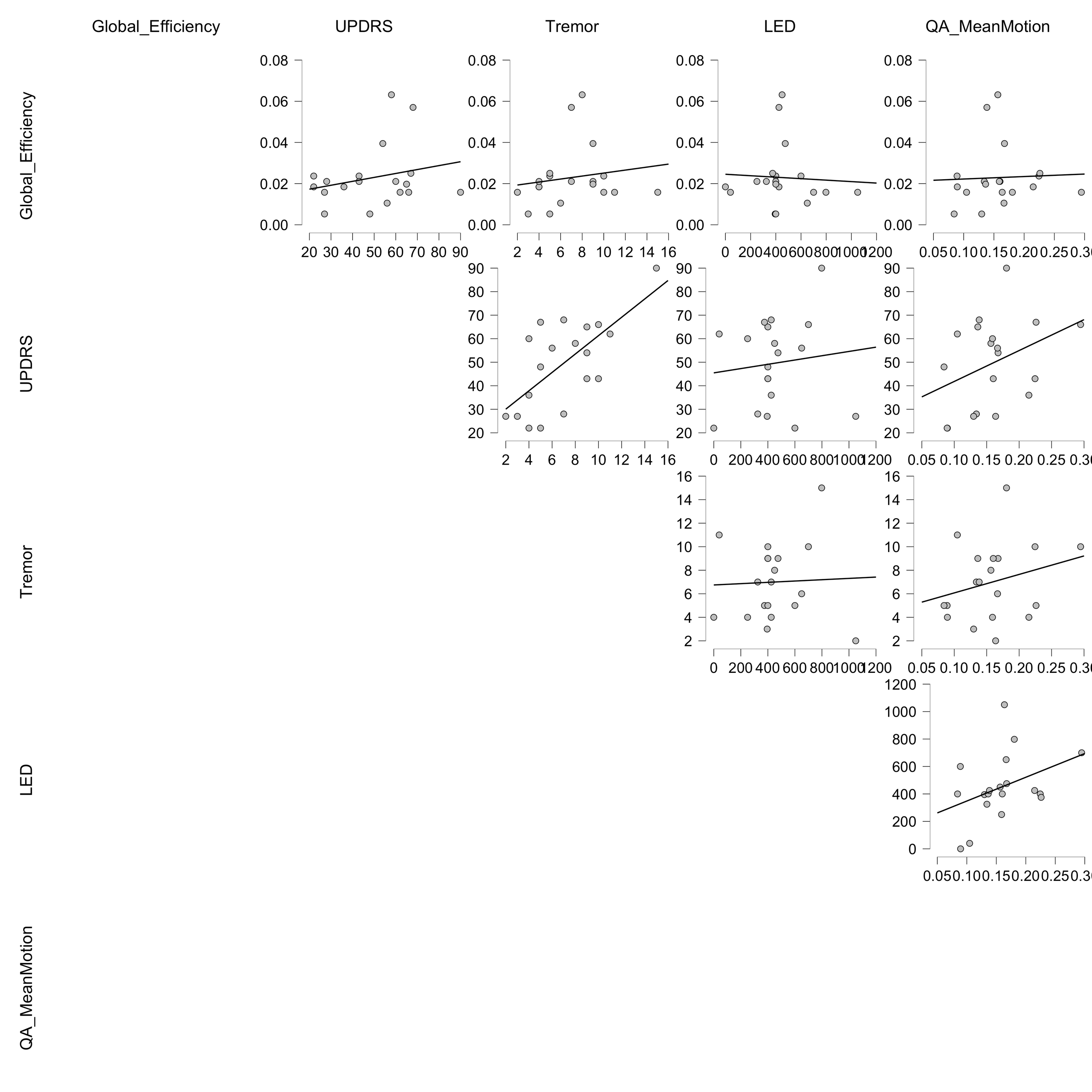


The following plots correspond to the PDAR group covariates correlation analyses.

## Correlation

| **Spearman's Correlations** | | | | | | | | | | | | | |
| --- | --- | --- | --- | --- | --- | --- | --- | --- | --- | --- | --- | --- | --- |
| **Variable** | |  | | **Global_Efficiency** | | **UPDRS** | | **Tremor** | | **LED** | | **QA_MeanMotion** | |
| Global_Efficiency |  | Spearman's rho |  | — |  |  |  |  |  |  |  |  |  |
|  |  | p-value |  | — |  |  |  |  |  |  |  |  |  |
| UPDRS |  | Spearman's rho |  | 0.287 |  | — |  |  |  |  |  |  |  |
|  |  | p-value |  | 0.175 |  | — |  |  |  |  |  |  |  |
| Tremor |  | Spearman's rho |  | 0.325 |  | 0.484 |  | — |  |  |  |  |  |
|  |  | p-value |  | 0.121 |  | 0.016 |  | — |  |  |  |  |  |
| LED |  | Spearman's rho |  | -0.019 |  | -0.046 |  | -0.006 |  | — |  |  |  |
|  |  | p-value |  | 0.928 |  | 0.832 |  | 0.977 |  | — |  |  |  |
| MeanMotion |  | Spearman's rho |  | -0.161 |  | -0.007 |  | 0.019 |  | -0.019 |  | — |  |
|  |  | p-value |  | 0.451 |  | 0.973 |  | 0.930 |  | 0.931 |  | — |  |
|  | | | | | | | | | | | | | |

### Correlation plot


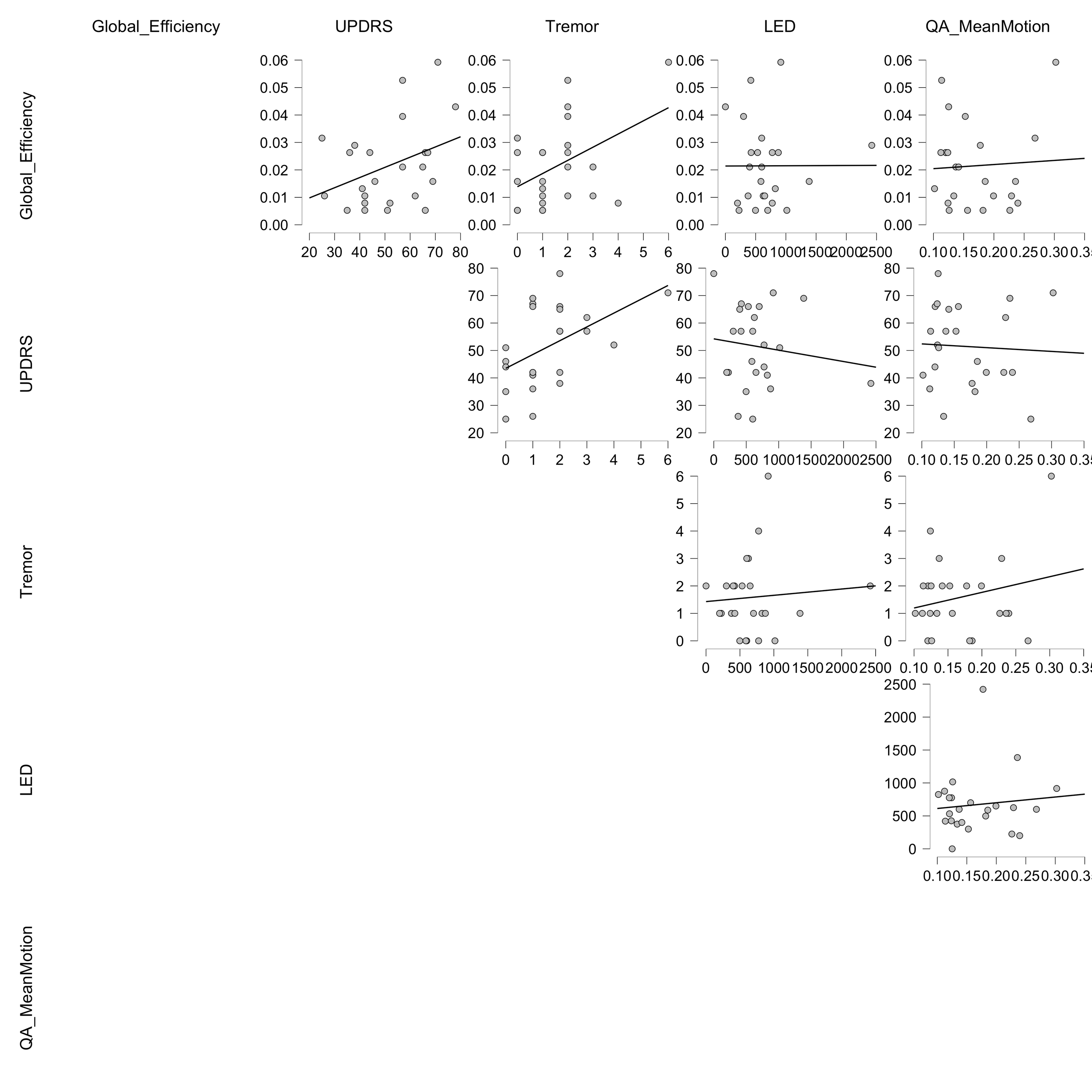

Supplement: Supplementary file 1 — Appendix S1 [file BRB3-11-e02178-s001.docx]
